# Sex difference in incidence and risk factors of hospitalization for heart failure, and subsequent mortality: findings from the China PEACE million persons project

**DOI:** 10.1186/s12889-023-17286-z

**Published:** 2023-11-28

**Authors:** Weida Qiu, Anping Cai, Zhiqiang Nie, Jiabin Wang, Yanqiu Ou, Yingqing Feng

**Affiliations:** 1 Department of Cardiology, Hypertension Research Laboratory, Guangdong Cardiovascular Institute, Guangdong Provincial People’s Hospital, Guangdong Academy of Medical Sciences, Guangzhou, China; 2 The Second School of Clinical Medicine, Southern Medical University, Guangzhou, China; 3 Global Health Research Center, Guangdong Provincial People ’ s Hospital, Guangdong Academy of Medical Sciences, Guangzhou, China

**Keywords:** Sex differences, Heart Failure, Mortality, Risk factors, Population

## Abstract

**Background:**

Epidemiological study of sex differences in incidence and risk factors of heart failure (HF), and subsequent mortality attributed to HF in the Chinese general population is lacking. This study aims to assess the sex differences in the incidence and risk factors of hospitalization for HF and evaluate the sex differences in population attributable fractions (PAFs) for the subsequent mortality among the general population.

**Methods:**

Data were from a sub-cohort of the Patient-Centered Evaluative Assessment of Cardiac Events Million Persons Project in China. Fine and Gray models were conducted to calculate the hazard ratios (HRs) and 95% confidence intervals (CIs) accounting for the competing risk of all-cause mortality. Propensity score matching analysis and subgroup analyses were used to verify the robustness of the results. Adjusted PAFs of HF for all-cause and cardiovascular mortality were evaluated by sex.

**Results:**

Of the 102,278 participants, 60.5% were women, and the mean age was 54.3 years. After a median follow-up of 3.52 years, 1588 cases of hospitalization for HF were identified. After adjusting for the covariates, women had 31% (95% CI: 0.61–0.79) lower risk for HF than men. The results were consistent in the propensity score matching cohort and across all subgroup analyses (all P sex-subgroups interaction > 0.05). Although women were associated with a lower risk of HF, they had a higher PAF (24.2%, 95% CI: 16.0-31.6) for subsequent cardiovascular mortality than men (16.5%, 95% CI: 11.3–21.5). Several significant differences in risk factors for HF were noted between sexes.

**Conclusion:**

In the southern Chinese population, women had a lower risk of HF but had a higher cardiovascular mortality fraction attributed to HF than men. Sex-specific preventative strategies and management for HF should be warranted.

**Supplementary Information:**

The online version contains supplementary material available at 10.1186/s12889-023-17286-z.

## Background

Striking sex differences in heart failure (HF) have been well-documented in Western countries, ranging from epidemiology and pathophysiological factors to therapeutic response and prognosis [1]. In Europe and the US, the prevalence of HF is fairly comparable between sexes [2–4], whereas women younger than 74 years of age have a significantly lower risk for incident HF than men [5]. Moreover, marked sex differences in risk factors, such as diabetes mellitus (DM) [6–8], hypertension [9], socioeconomic status (SES) [10, 11], tobacco smoking and alcohol consumption [12], for HF have also been well-described in western populations.

Epidemiologic studies of HF between sexes are relatively limited in Asia [13], especially in China. To date, only one previous study showed a similar weighted prevalence of HF among Chinese men and women (men versus women: 1.4% versus 1.2%) [14], and the sex difference in HF-related hospitalization in China is still unknown. Furthermore, sex differences in risk factors for HF and subsequent mortality attributed to HF in the Chinese population remain inconclusive [15]. Against the background of the high prevalence and heavy burden of HF in China [16], better clarifications of sex differences in incidence and risk factors of HF, and subsequent mortality due to HF are immediately needed, thus promoting sex-specific prevention and treatment strategies for HF.

Accordingly, leveraging a prospective sub-cohort of the China Patient-Centered Evaluative Assessment of Cardiac Events (PEACE) Million Persons Project, we aimed to (1) assess the sex differences in the incidence and risk factors of hospitalization for HF, and (2) evaluate the sex differences in population attributable fractions (PAFs) for the subsequent mortality among the Chinese general population.

## Materials and methods

### Study design and participant

The China PEACE Million Persons Project details have been described elsewhere [17, 18]. Briefly, this project is a nationwide, community-based cardiovascular disease (CVD) screening study that is aimed at identifying high CVD-risk individuals. Our current study was conducted in a sub-cohort of the China PEACE Million Persons Project, including 102,358 participants who were enrolled in 8 sites from Guangdong Province (Southern China) between January 2016 and December 2020. At each site, eligible participants aged 35 to 75 years with local residence registration were recruited by local staff after written informed consent was obtained. In the present study, we excluded 80 individuals with prevalent HF, and a total of 102,278 subjects were finally included.

### Data collection and variables

Information on sociodemographic characteristics (sex, age, resident area, married status, smoking and drinking status, education, occupation, annual household income, health insurance) and comorbid conditions (hypertension, DM, dyslipidemia, coronary artery disease (CAD), coronary revascularization, stroke, chronic obstructive pulmonary disease (COPD)) were collected by trained medical staff during face-to-face interviews. Each participant then received a physical examination to measure blood pressure, height, weight, and waist circumference using standard protocols. Seated blood pressure and pulse were measured on the participants’ right arm using an electronic blood pressure monitor (Omron HEM-7430; Omron Corporation, Kyoto, Japan) after 5 min of rest. Each subject’s blood pressure and pulse were measured twice at a 1-minute interval, and the mean value of 2 measurements was used. Height, weight, and waist circumference were measured by trained technicians, and individuals were required to wear light clothes without shoes and a cap. Body mass index (BMI) was calculated by dividing the weight in kilograms by the square of height in meters. Fingertip blood samples were used to test lipid profile (CardioChek PA Analyzer; Polymer Technology Systems, Indianapolis, Indiana, USA) and fasting blood glucose (FBG) (BeneCheck BK6–20 M Multi­Monitoring System, Suzhou Pu Chun Tang Biotechnology, China).

### Study outcome

The primary outcome of the current study was hospitalization for HF. Using the code of the Tenth Revision of the International Classification of Diseases (ICD-10) [19], participants’ HF-related hospitalization (I50) records were reviewed and identified from the Inpatients Registry by trained staff who were blinded to their baseline characteristics. HF was ascertained based on the presence of symptoms or signs (e.g., dyspnea, jugular vein distention, ankle swelling), the elevation of natriuretic peptide level, and the abnormality of cardiac function or structure on echocardiography. Finally, a panel of experienced experts consisting of 2 cardiologists and 1 statistician independently verified and ascertained all HF events. In addition, we calculated the PAFs for subsequent all-cause mortality and cardiovascular (CV) mortality due to HF among men and women. The events of death were collected from the China’s Centre for Disease Prevention and Control’s National Mortality Surveillance System and Vital Registration, and the ICD-10 was also used to code the all-cause and CV mortality records (I00-I99). The date of HF-related hospitalization, the death date, or the last follow-up date (December 31, 2021) was used to calculate the follow-up time, whichever came first.

### Statistical analysis

Continuous and categorical variables were presented as mean ± standard deviation (SD) and number (proportion) respectively. Differences in the baseline characteristics of participants between sexes were tested using Student’s t-test and χ^2^ test, accordingly. The Fine and Gray estimator was conducted to compute cumulative incidence curves for HF-related hospitalization and all-cause mortality before hospitalization for HF as a competing risk, and hazard ratios (HRs) and 95% confidence intervals (CIs) were calculated with adjustment for demographic and socioeconomic information, including age, smoking and drinking status, residential area, education, annual household income, and insurance (Model 1); Model 1 plus physical examination and laboratory, including systolic blood pressure (SBP), pulse, BMI, waist circumference, lipid profiles, and FBG (Model 2); Model 2 plus comorbidities, including hypertension, DM, dyslipidemia, CAD, coronary revascularization, stroke, and COPD (Model 3). Multiple imputations by chained equations with 20 imputations were used to fill in the missing values (< 1%). Several subgroup analyses were conducted and the sex-by-subgroup interactions were tested in Fine and Gray models based on likelihood ratio tests. Seven subgroups, including age (< 60 and ≥ 60 years), residential area (urban and rural), smoking status (current and non-current), education attainment (< high school and ≥ high school), annual household income (< 50,000 and ≥ 50,000 China Yuan), hypertension (yes and no), and DM (yes and no), were selected based on the following criteria: (1) A certain number of participants in each group; (2) Potential effect modifiers of the association between sex with hospitalization for HF.

To verify the robustness of the results, a 1:1 ratio propensity-matched analysis with a caliper value of 0.01 was performed. All 26 variables presented in Table 1 were selected for the propensity model to ensure the comparability of baseline characteristics between sexes. The propensity to men versus women was predicted by the multivariable logistic regression model, and post-estimations for propensity score-matched were evaluated by standardized bias and propensity score density [20]. Individuals were matched to the nearest available propensity score without replacement, and the procedure was repeated until all male participants were matched. The Fine and Gray models were then conducted in the propensity score-matched cohort.

**Table 1:** Baseline characteristics comparisons between men and women

Variable	Overall (N = 102,278)	Men (N = 40,393)	Women (N = 61,885)	P-value
**Demographic**				
Age (years)	54.3 ± 10.2	54.4 ± 10.4	54.2 ± 10.0	< 0.001
Age group, n(%)				< 0.001
35 ~ 44 years	20,508 (20.1)	8,364 (20.7)	12,144 (19.6)	
45 ~ 54 years	32,370 (31.7)	12,342 (30.6)	20,028 (32.4)	
55 ~ 64 years	29,920 (29.3)	11,420 (28.3)	18,500 (29.9)	
65 ~ 75 years	19,480 (19.1)	8,267 (20.5)	11,213 (18.1)	
Married, n(%)	92,524 (90.5)	37,474 (92.8)	55,050 (89.0)	< 0.001
Urban residence, n(%)	49,454 (48.4)	19,248 (47.7)	30,206 (48.8)	< 0.001
Current smoker, n(%)	17,586 (17.2)	17,158 (42.5)	428 (0.7)	< 0.001
Current drinker, n(%)	5,421 (5.3)	4,859 (12.0)	562 (0.9)	< 0.001
**Socioeconomic information**				
Education attainment ≥ high school, n(%)	30,268 (29.6)	15,030 (37.2)	15,238 (24.6)	< 0.001
Occupation, n(%)				< 0.001
Managers or professionals	8,502 (8.3)	4,601 (11.4)	3,901 (6.3)	
Agricultural, manufacturing, services or sales workers	40,345 (39.5)	16,606 (41.1)	23,739 (38.4)	
Housework, retired, unemployed or other occupations	53,431 (52.2)	19,186 (47.5)	34,245 (55.3)	
Annual household income ≥ 50,000 (CNY)	46,389 (45.4)	19,393 (48.0)	26,996 (43.6)	< 0.001
Health insurance, n(%)	95,388 (93.3)	37,904 (93.8)	57,484 (92.9)	< 0.001
**Physical examination**				
Systolic blood pressure (mmHg)	130.1 ± 19.0	131.6 ± 17.9	129.1 ± 19.6	< 0.001
Diastolic blood pressure (mmHg)	79.2 ± 11.3	81.7 ± 11.2	77.5 ± 11.1	< 0.001
Pulse (beat per minute)	77.4 ± 10.6	76.9 ± 11.0	77.7 ± 10.3	< 0.001
Body mass index (kg/m^2^)	24.1 ± 3.3	24.4 ± 3.2	24.0 ± 3.4	< 0.001
Waist circumference (cm)	83.6 ± 9.5	86.7 ± 9.1	81.5 ± 9.3	< 0.001
**Comorbidity**				
Hypertension, n(%)	23,167 (22.7)	10,090 (25.0)	13,077 (21.1)	< 0.001
Diabetes mellitus, n(%)	7,722 (7.6)	3,529 (8.7)	4,193 (6.8)	< 0.001
Dyslipidemia, n(%)	5,763 (5.6)	2,402 (6.0)	3,361 (5.4)	< 0.001
Coronary artery disease, n(%)	833 (0.8)	546 (1.4)	287 (0.5)	< 0.001
Coronary revascularization, n(%)	563 (0.6)	415 (1.0)	148 (0.2)	< 0.001
Stroke, n(%)	623 (0.6)	369 (0.9)	254 (0.4)	< 0.001
COPD, n(%)	136 (0.1)	98 (0.2)	38 (0.1)	< 0.001
**Laboratory**				
Total cholesterol (mmol/L)	4.91 ± 1.22	4.64 ± 1.17	5.09 ± 1.23	< 0.001
Triglyceride (mmol/L)	1.63 ± 0.97	1.72 ± 1.05	1.57 ± 0.90	< 0.001
LDL-C (mmol/L)	2.72 ± 1.00	2.58 ± 0.96	2.80 ± 1.01	< 0.001
HDL-C (mmol/L)	1.48 ± 0.44	1.32 ± 0.39	1.58 ± 0.43	< 0.001
Fasting blood glucose (mmol/L)	5.89 ± 1.68	5.96 ± 1.80	5.84 ± 1.59	< 0.001

CNY, China Yuan; COPD, chronic obstructive pulmonary disease; LDL-C, low density lipoprotein-cholesterol; HDL-C, high density lipoprotein cholesterol

To detect the risk factors for HF-related hospitalization among men and women, multivariate Cox regression analyses were performed between sexes, and the sex-by-factor interactions were also tested. To compute the PAFs for the subsequent all-cause and CV mortality attributed to HF, the ‘punaf’ command in STATA software was employed with further adjustment for the identical covariables included in the Fine and Gray model. This command implements the method for estimating PAFs for cross-section and cohort studies as recommended by Greenland S and Drescher K [21].

All analyses were performed using Stata MP version 17.1 (StataCorp LLC, College Station, TX, USA). Two-sided p-values < 0.05 were considered statistically significant.

## Results

### Baseline characteristics of participants

A total of 102,278 participants were enrolled, and 60.5% were women. The mean age was 54.3 years old, and the most prevalent comorbidity was hypertension (22.7%), followed by DM (7.6%) and dyslipidemia (5.6%). Compared with men, women were younger and less likely to smoke and drink and had lower SES (i.e., women had lower educational levels, lower annual household income, and were less likely to have health insurance). By contrast, men had higher comorbid burdens compared to women. (Table 1)

The baseline characteristics of participants with and without hospitalization for HF were also presented in Supplemental Table 1. Briefly, compared with individuals without HF-related hospitalization, those with HF were older and more likely to be smokers, had lower SES, had higher levels of baseline blood pressure and pulse, were more obese, and had higher comorbid burdens.

### Sex differences in hospitalization for heart failure

**Fig. 1:**
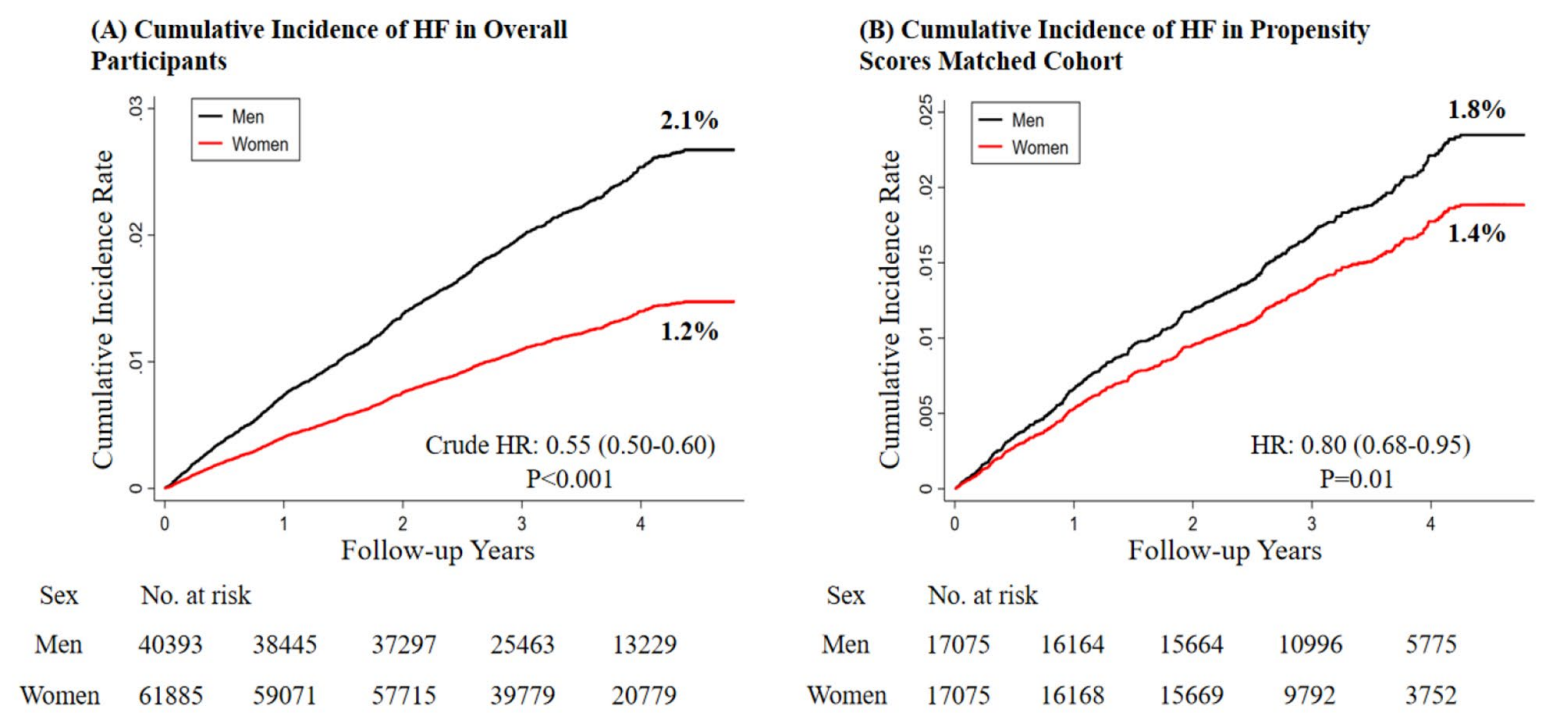
Cumulative Incidence Curves of Hospitalization for Heart failure in Overall (A) and Propensity Scores Matched (B) Cohort. HF, heart failure, HR, hazard ratio

After a median follow-up of 3.52 years (343,758 person-years), 1588 cases of hospitalization for HF (4.62 (95% CI: 4.40–4.85) per 1000 person-year) were identified. The incidence rate for men and women was 6.37 (95% CI: 5.95–6.80) and 3.49 (95% CI: 3.24–3.75) per 1000 person-year, respectively (p for incidence rate comparison < 0.001). The cumulative rate of HF-related hospitalization was lower in women than in men (Fig. 1 Panel A), with an adjusted HR of 0.69 (95% CI: 0.61–0.79). (Table 2)

**Table 2:** Association between sex and hospitalization for heart failure in all participants and in propensity scores matched participants

Overall Participants				
	Overall (N = 102,278)	Men (N = 40,393)	Women (N = 61,885)	P-value
Time (person-years)	34,3758	13,5199	20,8559	/
Event (%)	1,588 (1.6)	861 (2.1)	727 (1.2)	< 0.001
Incidence rate (per 1000 person-year)	4.62 (4.40–4.85)	6.37 (5.95–6.80)	3.49 (3.24–3.75)	< 0.001
Crude (HR and 95% CI)	/	1 (Reference)	0.55 (0.50–0.60)	< 0.001
Model 1 (HR and 95% CI)	/	1 (Reference)	0.63 (0.56–0.71)	< 0.001
Model 2 (HR and 95% CI)	/	1 (Reference)	0.67 (0.59–0.77)	< 0.001
Model 3 (HR and 95% CI)	/	1 (Reference)	0.69 (0.61–0.79)	< 0.001
**Propensity Scores Matched Participants**				
	**Overall (N = 34,150)**	**Men (N = 17,075)**	**Women (N = 17,075)**	**P-value**
Time (person-years)	11,1448	57,237	54,211	/
Event (%)	530 (1.6)	314 (1.8)	216 (1.3)	< 0.001
Incidence rate (per 1000 person-year)	4.76 (4.37–5.18)	5.49 (4.91–6.13)	3.98 (3.49–4.55)	< 0.001
h and 95% CI	/	1 (Reference)	0.72 (0.61–0.86)	< 0.001

Model 1 adjusted for age, smoking and drinking status, residential area, education, annual household income, and insurance;

Model 2 adjusted model 1 plus systolic blood pressure, pulse, body mass index, waist circumference,total cholesterol, triglyceride, low density lipoprotein-cholesterol, high density lipoprotein cholesterol, and fasting blood glucose ;

Model 3 adjusted for model 2 plus hypertension, diabetes mellitus, dyslipidemia, coronary artery disease, coronary revascularization, stroke, and chronic obstructive pulmonary disease

HR, hazard ratio; CI, confidence interval

Due to the significant differences in baseline characteristics between sexes, we performed the propensity-matched analysis. We matched 17,075 men to women in a 1:1 ratio, and the differences and the standardized bias significantly reduced between groups after propensity matching. (Supplemental Figs. 1 and 2, Supplemental Table 2) The lower risk of hospitalization for HF observed in women remained consistent in the propensity matching analysis (HR: 0.72, 95% CI: 0.61–0.86). (Fig. 1 Panel B, Table 2)

**Fig. 2:**
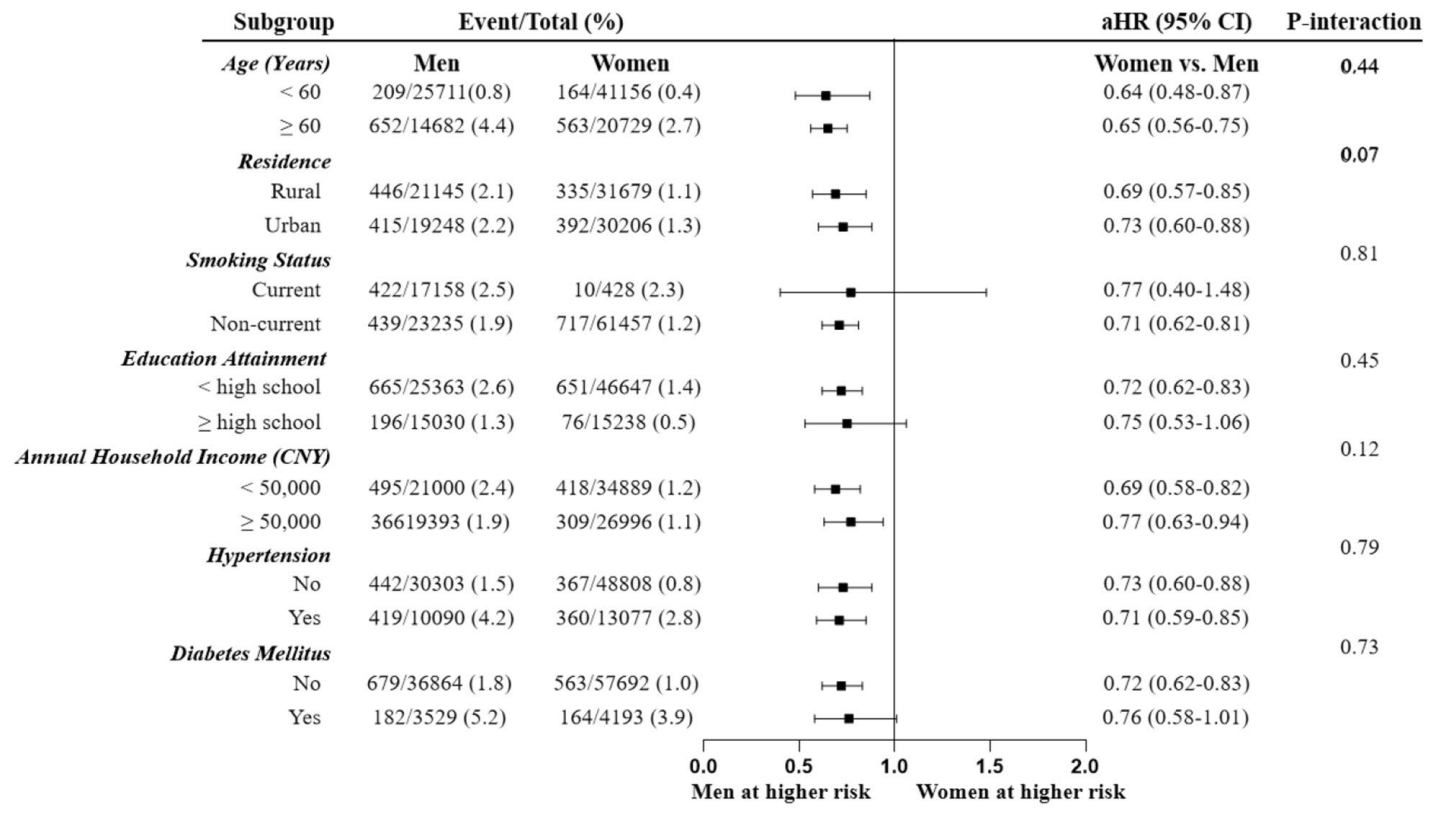
Subgroup analyses. CNY, China Yuan

In the several subgroup analyses, including age (higher or lower than 60 years old), residence area (urban or rural), smoking status (current or non-current), education attainment (higher or lower than high school), annual household income (higher or lower than 50,000 Chinese Yuan), hypertension (yes or no), and DM (yes or no), women consistently had a lower risk of HF in comparison with men (all p-interaction > 0.05). (Fig. 2)

### Sex differences in risk factors for heart failure

SBP, pulse, waist circumference, hypertension, DM, CAD, COPD, and FBG were the significant independent risk factors for HF in both men and women. Among these risk factors, higher age, waist circumference, and triglyceride had a more pronounced impact on HF development in women than in men (p for sex-factor interactions < 0.05). Additionally, urban residence, higher annual household income, and prevalent stroke were also associated with a higher risk of HF in women. (Table 3)

**Table 3:** Factors associated with hospitalization for heart failure in men and women

	Men (HR & 95% CI)	Women (HR & 95% CI)	Women:Men Relative Risk Ratio (95% CI)	P-interaction
Age (per 1-SD increment)	**2.44 (2.21–2.70)**	**2.96 (2.64–3.32)**	**1.16 (1.02–1.32)**	**0.03**
Rural residence (vs. urban)	1.11 (0.96–1.28)	**1.31 (1.12–1.53)**	**1.19 (1.02–1.36)**	**0.04**
Current smoker (vs. Non-current)	**1.45 (1.26–1.68)**	1.77 (0.92–3.39)	1.23 (0.65, 2.31)	0.40
Education attainment (vs. <high school)	**0.79 (0.66–0.94)**	**0.72 (0.56–0.94)**	0.90 (0.61–1.24)	0.19
Annual household income (vs. <50,000 CNY)	1.00 (0.86–1.15)	**1.23 (1.05–1.43)**	**1.25 (1.02–1.55)**	**0.04**
Systolic blood pressure (per 1-SD increment)	**1.13 (1.03–1.25)**	**1.11 (1.01–1.23)**	1.00 (0.99–1.01)	0.15
Pulse (per 1-SD increment)	**1.13 (1.06–1.20)**	**1.08 (1.01–1.23)**	0.97 (0.89–1.07)	0.47
Waist circumference (per 1-SD increment)	**1.25 (1.11–1.40)**	**1.44 (1.29–1.61)**	**1.22 (1.10–1.34)**	**0.001**
Hypertension (vs. No)	**1.62 (1.38–1.89)**	**1.50 (1.26–1.78)**	0.91 (0.72–1.18)	0.33
Diabetes mellitus (vs. No)	**1.55 (1.27–1.90)**	**1.62 (1.30–2.01)**	1.07 (0.93–1.34)	0.13
Coronary artery disease (vs. No)	**3.66 (2.04–6.57)**	**3.13 (1.47–6.64)**	0.87 (0.61–1.43)	0.72
Stroke (vs. No)	0.60 (0.32–1.12)	**2.17 (1.23–3.83)**	**3.46 (1.56–7.69)**	**0.001**
COPD (vs. No)	**4.28 (2.28–8.03)**	3.92 (0.91–16.92)	0.94 (0.21–4.23)	0.85
Triglyceride (per 1mmol/L increment)	1.05 (0.95–1.16)	**1.18 (1.09–1.28)**	**1.12 (1.02–1.23)**	**0.01**
HDL-C (per 1mmol/L increment)	**1.42 (1.13–1.78)**	1.14 (0.91–1.45)	0.83 (0.68–1.09)	0.34
Fasting blood glucose (per 1mmol/L increment)	**1.06 (1.02–1.09)**	**1.06 (1.03–1.10)**	1.01 (0.97–1.06)	0.20

h, hazard ratio; CI, confidence interval; SD, standardized difference; CNY, China Yuan; COPD, chronic obstructive pulmonary disease; HDL-C, high density lipoprotein cholesterol

The **bold font** indicates significance

### Sex difference in population attributable fraction for mortality attributed to heart failure

**Fig. 3:**
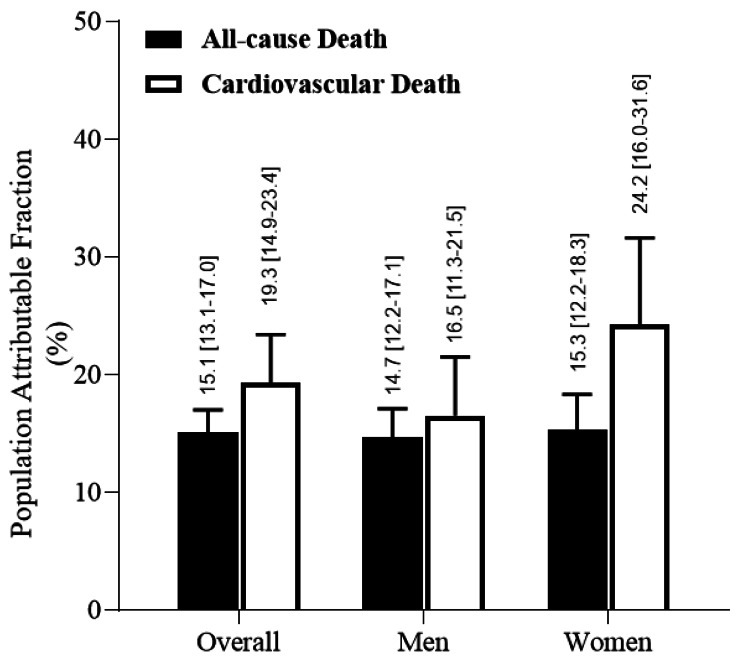
Population Attributable Fraction of Heart Failure for All-cause and Cardiovascular Death Stratified by Sex

The overall PAFs for subsequent all-cause mortality and CV mortality attributed to HF were 15.1% (95% CI: 13.7–17.0) and 19.3% (95% CI: 14.9–23.4), respectively. Specifically, the PAFs for all-cause mortality were comparable between men (14.7%, 95% CI: 12.2–17.1) and women (15.3%, 9%% CI: 12.2–18.3), while the PAF for CV mortality was higher in women (24.2%, 95% CI: 16.0-31.6) than in men (16.5%, 95% CI: 11.3–21.5). (Fig. 3)

## Discussion

In this large, prospective, population-based cohort study, women had a lower risk of hospitalization for HF than men, and the results remained consistent in the conventional risk factors that were adjusted, in the propensity-matched analysis as well as in the subgroup analyses. Majorities of modifiable risk factors were associated with HF both in men and women, several sex differences in risk factors (e.g., age, waist circumference) were also found. Although female sex was associated with a lower HF risk, women had a higher PAF for subsequent CV mortality than men.

Sex differences in the incidence of HF have been well-documented in Western countries. A recent study including over 70,000 Europeans from 4 community-based cohorts found women had a lower risk for incident HF than men, with fewer HF cases noted in women (5.9%) than in men (7.3%) after a median follow-up of 12.7 years [22]. Another pooled study consisting of 12,417 community-dwelling adults from the U.S. also showed a higher lifetime risk for HF in men (27.4%) than in women (23.8%) [23]. Interestingly, more and more studies demonstrated that men developed HF earlier than women in middle-aged (< 75 years) whereas women had a higher incidence rate of HF in old-aged [5, 24]. However, little evidence exists regarding the sex difference in hospitalization for HF in China. Our current study is the first to describe the incidence of HF-related hospitalization by sexes in southern China. Consistent with the prior studies from Western countries, our study showed a lower risk for HF was observed in women aged 35 to 75 years, and the conclusion remained robust in the propensity-matched analysis, suggesting intensive and improved preventative strategies should be warranted in middle-aged men.

In line with previous studies [12, 25], the majority of conventional risk factors were related to HF-related hospitalization in both sexes in the present study, including age, lower education attainment, higher levels of SBP, resting pulse, and waist circumference, hypertension, DM, CAD, and COPD. Of note, advanced age had a more pronounced impact on HF development in women than men, and this could be a reasonable explanation for why women have a higher incidence rate of HF than men in older age [5], indicating improved management of CV health should be considered in elderly women. In addition to age, women with greater waist circumference were predisposed to HF compared to men. A previous study also showed that obese women harbored a greater risk of future HF than men, especially in HF with preserved ejection fraction (HFpEF) [7], and the underlying mechanism may be due to the differential effect of abdominal obesity on longitudinal changes in left ventricular mass among women than men [26]. All in all, the current study demonstrated that the majority of modifiable risk factors tended to have more pronounced impacts on HF in women than men, although women had a lower risk of HF development.

Another previous study from the Western population demonstrated the differential association between SBP [22] and DM [12] with incident HF among men and women. In contrast to these studies, the current study showed that SBP, hypertension, and DM portended similar risks of HF between sexes in the Chinese general population. The difference might be mainly due to the distinct races enrolled in each study, and further studies including different racial groups are warranted to investigate the sex differences in risk factors for HF development. Nevertheless, immediate efforts are needed to ease the burden of the high incidence of HF in China [16] by emphasizing the management and prevention of chronic diseases (i.e., hypertension, DM, CAD).

Sex differences in mortality risk after incident HF are complicated and mixed. In the Framingham Heart Study (FHS) from 1990 to 1999, men with HF (59%) were associated with a higher 5-year all-cause mortality risk than their female counterparts (45%) [27]. By contrast, in the Olmsted County study from 2000 to 2010, the age-adjusted all-cause mortality risk was comparable between men and women (men vs. women: HR: 1.09, 95% CI: 0.99–1.20), whereas the lower CV mortality rate was noted in women (men vs. women: HR: 1.19, 95% CI: 1.03–1.37) [28]. In a study consisting of Asian individuals, women with acute HF had a 14% (95% CI: 0.79–0.94) lower mortality risk than men at 1-year follow-up [29]. Nevertheless, another study from the ASIAN-HF cohort showed that women with HF with reduced heart failure (HFrEF) patients and DM had a higher risk of 1-year all-cause mortality and HF hospitalization than men [30]. Our current study supports and extends the prior findings. In the general population, although female sex was associated with a lower risk of hospitalization for HF, women had a similar PAF for subsequent all-cause mortality and significantly higher PAF for subsequent CV mortality than men, indicating a worse prognosis in women with HF. The poor outcomes observed in women might be the consequence of the huge sex gap in standardized therapy of CV diseases [31–34]. More attention should be paid to female HF patients for the prevention of mortality.

Understanding the sex differences in risk factors for HF and subsequent mortality has significant public health implications for cardiovascular health promotion. First, most conventional risk factors were significantly associated with HF in both sexes, underlining the early intervention of these risk factors is of great significance to HF prevention at the population level. Second, an urgent call to action is needed to promote CV health in elderly women, not only the higher incidence of HF among older women [5, 24] but also the worse life quality observed in female HF patients [35]. Third, the majority of modifiable harbored a hazardous impact on hospitalization for HF in women than men, highlighting that primary care physicians should pay more attention to improving the management of common and conventional risk factors for women. Fourth, lower SES (i.e., rural residence, lower annual household income) has a stronger strength of association with female CV health than males. Nevertheless, gender inequality consistently exists and women always tend to have a poor SES, resulting in under-management of CVDs [31] as well as under-recognition of cardiovascular risk factors [36], eventually leading to a poor prognosis among women. Therefore, our study underscores the improvement of female SES, which might be a potential approach and an extremely important step to promote the CV health of women.

### Limitations

The present study has several noteworthy limitations. First, the current study was conducted in a sub-cohort of the China PEACE Million Persons Project from southern China, and the results should be extrapolated with caution to other regions with different lifestyles and genetic backgrounds. Second, due to the observational nature, unmeasured and missing confounding factors may still exist and influence the current results, such as the important laboratory indicators relative to cardiac injury and function, although we have adjusted for multiple conventional covariates and conducted the propensity-matched analysis. Third, the China PEACE Million Persons Project only performs echocardiography with high-CVD risk individuals, we cannot classify HF subtypes based on left ventricular ejection fraction (LVEF). Because patients with HFpEF are often undetected, particularly in the outpatient [37], and because women are susceptible to HFpEF [38], an underdiagnosis of HFpEF in women might be possible. Nevertheless, HF as an endpoint obtained from administrative registers has been demonstrated can be used in a cohort study with high specificity [39]. Fourth, given the epidemiologic nature of the current study, the underlying pathophysiological mechanisms of the sex differences in HF and risk factors cannot be fully explained.

### Future research

Future research could analyze the sex disparities in the incidence and risk factors of different HF subtypes according to LVEF (i.e., HFrEF, heart failure with mildly reduced LVEF, and HFpEF) using the nationally represented population. In addition, sex differences in mortality among Western HF patients have been reported [38], however, sex differences in prognosis of HF are still inconclusive and remain to be elucidated. What’s more, comparisons of the above sex differences between different regional and racial groups need to be considered, as significant regional and ethical differences in HF have been recently recorded [40, 41]. Lastly, as mentioned above, the underlying mechanisms of the sex differences in HF cannot be completely explained due to the observational and epidemiologic nature of the present study, which merits further study.

## Conclusions

Our large, population-based data provide evidence showing that female sex was associated with a lower risk of HF than men in the general population from southern China, and the results remained consistent in the propensity-matched analysis and across all the subgroups. Majorities of conventional risk factors were related to HF development in both sexes, whereas several significant sex differences in modifiable risk factors for HF were also noted, suggesting sex-specific preventative strategies for HF should be warranted. Although female sex was associated with a lower risk of HF-related hospitalization, women had a similar PAF for subsequent all-cause mortality and significantly higher PAF for subsequent CV mortality than men, indicating more attention should be paid to female HF patients for standardized management.

### Electronic supplementary material

Below is the link to the electronic supplementary material.

Supplementary Material 1

## Data Availability

The deidentified participant data will be shared on a request basis. Please directly contact the corresponding author to request data sharing.

## List of abbreviations

HF: heart failure
DM: diabetes mellitus
SES: socioeconomic status
PEACE: Patient-Centered Evaluative Assessment of Cardiac Events
PAF: population attributable fraction
CVD: cardiovascular disease
CAD: coronary artery disease
COPD: chronic obstructive pulmonary disease
BMI: body mass index
FBG: fasting blood glucose
ICD-10: the Tenth Revision of International Classification of Diseases
CV: cardiovascular
SD: standard deviation
HR: hazard ratio
CI: confidence interval
SBP: systolic blood pressure
HFpEF: heart failure with preserved ejection fraction
FHS: Framingham Heart Study
HFrEF: heart failure with reduced heart failure
