# Supplementary material for: Sex difference in incidence and risk factors of hospitalization for heart failure, and subsequent mortality: findings from the China PEACE million persons project
Source: BMC Public Health. 2023 Nov 28;23:2356. doi: 10.1186/s12889-023-17286-z (PMC10685651; doi:10.1186/s12889-023-17286-z)

Supplemental Table 1. Baseline characteristics comparisons between participants with and without incident heart failure.

Variable	Without incident HF (N=100,690)	With incident HF (N=1,588)	P-value
**Demographic**			
Male, n(%)	39,532 (39.3)	861 (54.2)	<0.001
Age (years)	54.1±10.1	64.2±7.9	<0.001
Age group, n(%)			<0.001
35~44 years	20,473 (20.3)	35 (2.2)	
45~54 years	32,213 (32.0)	157 (9.9)	
55~64 years	29,398 (29.2)	522 (32.9)	
65~75 years	18,606 (18.5)	874 (55.0)	
Married, n(%)	91,117 (90.5)	1,407 (88.6)	0.01
Urban residence, n(%)	48,647 (48.3)	807 (50.8)	0.05
Current smoker, n(%)	17,154 (17.0)	432 (27.2)	<0.001
Current drinker, n(%)	5,323 (5.3)	98 (6.2)	0.12
**Socioeconomic information**			
Education attainment ≥high school, n(%)	29,996 (29.8)	272 (17.1)	<0.001
Occupation, n(%)			<0.001
Managers or professionals	8,459 (8.4)	43 (2.7)	
Agricultural, manufacturing, services or sales workers	39,937 (39.7)	408 (25.7)	
Housework, retired, unemployed or other occupations	52,294 (51.9)	1,137 (71.6)	
Annual household income ≥ 50,000 (CNY)	45,714 (45.4)	675 (42.5)	0.02
Health insurance, n(%)	93,889 (93.3)	1,499 (94.4)	0.07
**Physical examination**			

Systolic blood pressure (mmHg)	129.9±19.0	138.8±20.4	<0.001
Diastolic blood pressure (mmHg)	79.1±11.3	80.1±12.5	<0.001
Heart rate (beat per minute)	77.3±10.5	78.3±11.9	<0.001
Body mass index (kg/m^2)	24.1±3.3	24.7±3.8	<0.001
Waist circumference (cm)	83.5±9.5	87.1±10.8	<0.001
**Comorbidity**			
Hypertension, n(%)	22,388 (22.2)	779 (49.1)	<0.001
Diabetes mellitus, n(%)	7,376 (7.3)	346 (21.8)	<0.001
Dyslipidemia, n(%)	5,609 (5.6)	154 (9.7)	<0.001
Coronary artery disease, n(%)	727 (0.7)	106 (6.7)	<0.001
Coronary revascularization, n(%)	479 (0.5)	84 (5.3)	<0.001
Stroke, n(%)	598 (0.6)	25 (1.6)	<0.001
COPD, n(%)	121 (0.1)	15 (0.9)	<0.001
**Laboratory**			
Total cholesterol (mmol/L)	4.91±1.22	4.86±1.46	0.06
Triglyceride (mmol/L)	1.63±0.96	1.82±1.05	<0.001
LDL-C (mmol/L)	2.72±1.00	2.63±1.17	0.001
HDL-C (mmol/L)	1.48±0.44	1.44±0.45	0.001
Fasting blood glucose (mmol/L)	5.88±1.65	6.65±2.62	<0.001

CNY, China Yuan; COPD, chronic obstructive pulmonary disease; LDL-C, low density lipoprotein-cholesterol; HDL-C, high density lipoprotein-cholesterol

Supplemental Table 2. Baseline characteristics comparisons between men and women in the propensity-matched participants.

Variable	Men (N=17,075)	Women (N=17,075)	Standardize bias (%)
**Demographic**			
Age (years)	54.5±10.8	54.7±10.2	1.7
Married, n(%)	15,691 (91.9)	15,770 (92.4)	1.6
Urban residence, n(%)	8,242 (48.3)	8,333 (48.8)	1.1
Current smoker, n(%)	459 (2.7)	406 (2.4)	0.9
Current drinker, n(%)	422 (2.5)	430 (2.5)	0.2
**Socioeconomic information**			
Education attainment ≥high school, n(%)	5,968 (35.0)	6,114 (35.8)	1.9
Occupation, n(%)			0.9
Managers or professionals	1,584 (9.3)	1,858 (10.9)	
Agricultural, manufacturing, services or sales workers	7,048 (41.3)	6,595 (38.6)	
Housework, retired, unemployed or other occupations	8,443 (49.5)	8,622 (50.5)	
Annual household income ≥ 50,000 (CNY)	7,877 (46.1)	7,871 (46.1)	0.1
Health insurance, n(%)	15,841 (92.8)	15,798 (92.5)	1.0
**Physical examination**			
Systolic blood pressure (mmHg)	131.2±17.6	131.5±19.9	1.6
Diastolic blood pressure (mmHg)	80.6±10.5	80.9±11.4	3.0
Heart rate (beat per minute)	76.8±11.0	76.7±9.9	0.8
Body mass index (kg/m^2)	24.3±3.1	24.2±3.3	1.5
Waist circumference (cm)	85.2±8.6	85.3±9.1	1.1
**Comorbidity**			
Hypertension, n(%)	4,210 (24.7)	4,182 (24.5)	0.4

Diabetes mellitus, n(%)	1,383 (8.1)	1,406 (8.2)	0.5
Dyslipidemia, n(%)	956 (5.6)	954 (5.6)	0.1
Coronary artery disease, n(%)	132 (0.8)	145 (0.9)	0.8
Coronary revascularization, n(%)	87 (0.5)	99 (0.6)	0.9
Stroke, n(%)	115 (0.7)	142 (0.8)	2.0
COPD, n(%)	16 (0.1)	24 (0.1)	1.2
**Laboratory**			
Total cholesterol (mmol/L)	4.73±1.11	4.72±1.05	0.9
Triglyceride (mmol/L)	1.58±0.83	1.57±0.79	0.6
LDL-C (mmol/L)	2.63±0.97	2.63±0.94	0.7
HDL-C (mmol/L)	1.38±0.38	1.38±0.35	0.8
Fasting blood glucose (mmol/L)	5.93±1.74	5.93±1.64	0.1

CNY, China Yuan; COPD, chronic obstructive pulmonary disease; LDL-C, low density lipoprotein-cholesterol; HDL-C, high density lipoprotein cholesterol

Supplemental Figure 1. Standardized Bias across Covariates Before and After Propensity-matched.

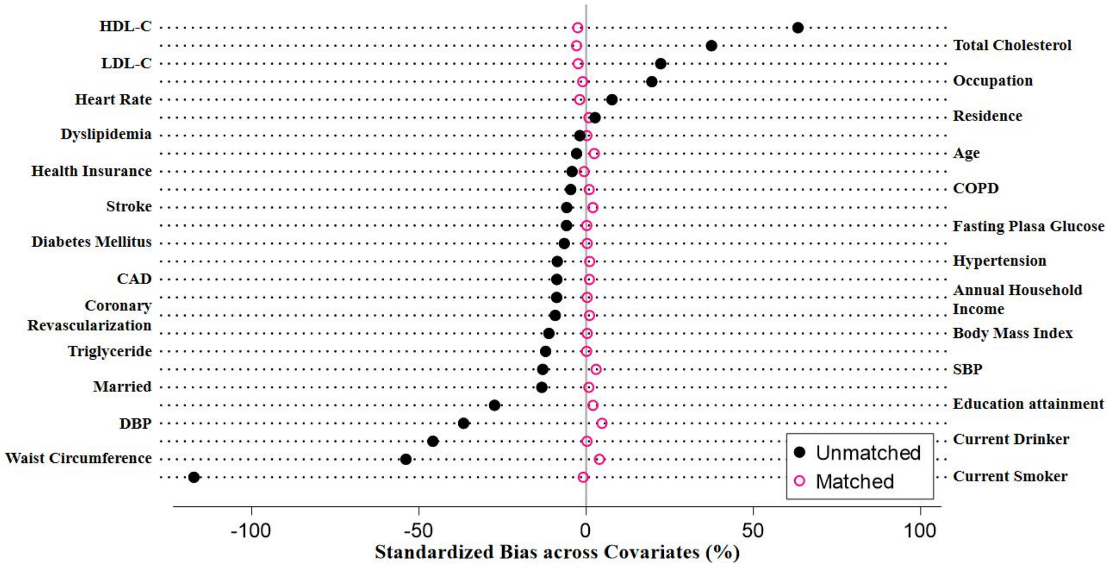

LDL-C, low-density lipoprotein cholesterol; COPD, chronic obstructive pulmonary disease; CAD, coronary artery disease; SBP, systolic blood pressure; DBP, diastolic blood pressure.

Supplemental Figure 2. Estimated Density of The Propensity Scores Before and After Propensity Scores Matched.

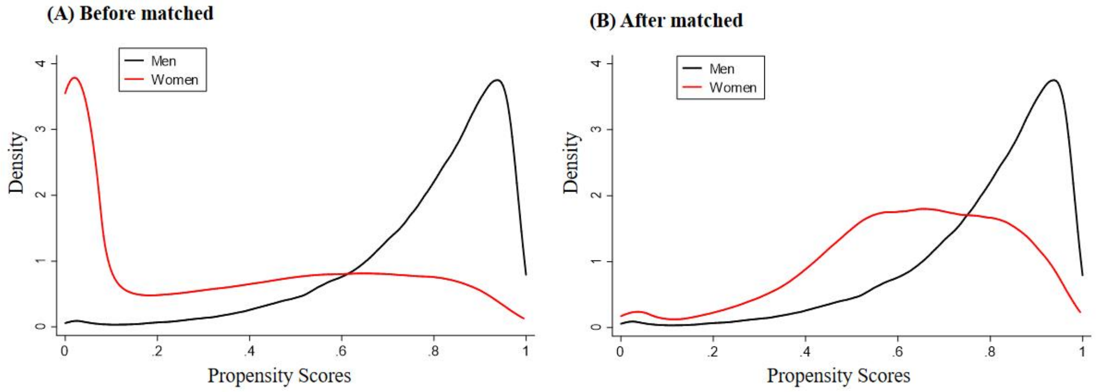

Supplement: Supplementary file 1 — Supplementary Material 1 [file 12889_2023_17286_MOESM1_ESM.pdf]
